# Detection of Explosives by SERS Platform Using Metal Nanogap Substrates

**DOI:** 10.3390/s21165567

**Published:** 2021-08-18

**Authors:** Samir Adhikari, Emmanuel K. Ampadu, Minjun Kim, Daegwon Noh, Eunsoon Oh, Donghan Lee

**Affiliations:** Department of Physics, Chungnam National University, Daejeon 34134, Korea; frendsamir@cnu.ac.kr (S.A.); ekampadu@cnu.ac.kr (E.K.A.); kmjun1674@cnu.ac.kr (M.K.); fo1109@cnu.ac.kr (D.N.)

**Keywords:** nitro compounds, explosives detection, SERS, nanogap, surface plasmon, field enhancement

## Abstract

Detecting trace amounts of explosives to ensure personal safety is important, and this is possible by using laser-based spectroscopy techniques. We performed surface-enhanced Raman scattering (SERS) using plasmonic nanogap substrates for the solution phase detection of some nitro-based compounds, taking advantage of the hot spot at the nanogap. An excitation wavelength of 785 nm with an incident power of as low as ≈0.1 mW was used to excite the nanogap substrates. Since both RDX and PETN cannot be dissolved in water, acetone was used as a solvent. TNT was dissolved in water as well as in hexane. The main SERS peaks of TNT, RDX, and PETN were clearly observed down to the order of picomolar concentration. The variations in SERS spectra observed from different explosives can be useful in distinguishing and identifying different nitro-based compounds. This result indicates that our nanogap substrates offer an effective approach for explosives identification.

## 1. Introduction

Explosive materials typically contain nitro compounds such as nitroaromatics (TNT), nitramines (RDX), and nitrate esters (PETN). Nitro compounds are commonly used for various military and civil purposes such as weapons and landmines, explosives for mining purposes, and as agricultural fertilizers. These compounds are often hazardous to both humans and environments, and thus, it is very important to find ways for the fast detection of these chemicals [1,2]. Although different sensor concepts such as ion mobility spectrometer and fluorescence quenching method [3,4] have been developed for analyzing nitro explosives, SERS (surface-enhanced Raman scattering) is one of the most powerful and versatile techniques [5,6,7,8,9,10,11,12].

Similar to the conventional Raman spectroscopy, SERS detects inelastic scattering of incident light via molecular vibrations [13]. While the Raman technique typically suffers from low sensitivity due to the limited number of scattered photons, SERS offers greatly amplified signals by several orders of magnitude [14,15]. In SERS, local electric fields are enhanced in the vicinity of metal nanostructures due to “trapping” of light or the surface plasmon effect. The location of the maximum electric field is called a hot spot [16], and the signal from an analyte molecule located at the hot spot can be substantially amplified. In addition, the physical or chemical adsorption of the analyte onto the surface of the nanostructures may improve the SERS signal [17,18]. Since the background noise as well as signal intensity increases with excitation power, it is preferable to obtain a sufficient signal intensity with a low excitation density by utilizing suitable nanostructures.

Kneipp reported SERS spectra from TNT on colloidal silver and gold particles for the first time in 1995 [19]. Since then, several commercially available SERS substrates as well as custom-made substrates have been used for the purpose of explosives detection [20,21,22,23,24]. Klarite substrates, composed of an array of inverted pyramidal-shaped pits etched into silicon, are the most well-characterized commercial substrate for SERS [25]. In the substrates, the hot spots are located at the bottom of the gold-coated pyramid wells and at the four sides. Botti et al. used Klarite substrates and achieved an LOD (limit of detection) of 5 pg PETN, 80 pg RDX, and 20 pg TNT [20]. On the other hand, Ko et al. obtained an LOD of 15–30 molecules of TNT by utilizing custom-made 3D nanopores [11].

In the case of NO_2_ molecules, the most distinct vibrational mode present in a Raman spectrum is a symmetric stretching mode at ≈1365 cm^−1^ [26]. If a nitro group is attached to an aromatic ring as in TNT, this vibrational mode shifts to a slightly lower frequency [27]. In the case of TNT molecules, the reported frequencies of NO_2_ symmetric stretching modes vary between 1330 and 1380 cm^−1^ in the literature [28,29,30,31,32], and the spectral shapes appear to be different from one another, which are not currently well understood.

**Figure 1:**
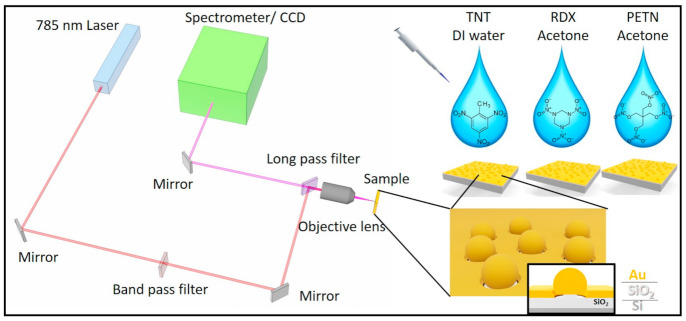
Schematic diagram of an experimental set-up.

Though very sensitive, serious problems with SERS signals are stability, reproducibility, and insufficient performance during practical applications, which may be due to the weak interaction between molecules and sensing substrates [24,33,34]. Recently, we have reported highly sensitive and uniform SERS substrates fabricated through simple processes of e-beam evaporation, rapid thermal annealing, and wet chemical etching [35]. The 6-inch SERS substrates are composed of uniformly distributed Au hole–sphere nanogaps (see Figure 1), which are highly inert to environments.

In this paper, we report on the SERS detection of three nitro-based explosives (TNT, RDX, and PETN) by employing our Au nanogap substrates. We were able to detect only a few molecules with an excitation power of as low as 0.1 mW. We also discuss the characteristic vibrational modes of these molecules and identify two distinct NO_2_ symmetric stretching modes for TNT molecules based on Density Functional Theory (DFT) simulations.

## 2. Materials and Methods

**Figure 2:**
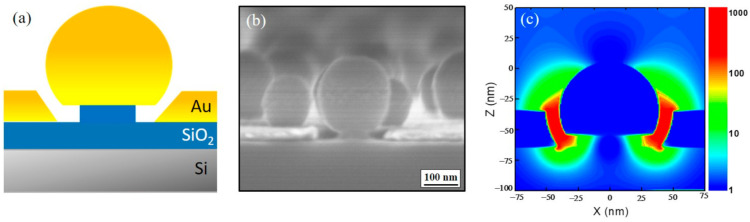
(**a**) Schematic illustration and (**b**) SEM image of an Au nanogap sample. (**c**) FDTD simulation result showing the enhancement of |E|^2^ around the nanogap in log scale, where E is the electric field.

Figure 1 shows the schematic diagram of a SERS experimental set-up. Au nanogap substrates were fabricated according to the procedures reported in [35]. Figure 2a shows a schematic picture of a gold nanogap. The substrate contains a metallic nanosphere standing on a SiO_2_ nanopillar and nanogap around the nanosphere. Localized surface plasmon resonance (LSPR) depends on the diameter of the nanosphere as well as the nanogap size, and thus, the resonant wavelength can be controlled by changing these parameters [36,37]. A fabricated 6-inch SERS substrate contains a high number of electromagnetic hot spots and can be sliced easily into small pieces for multiple experimental purposes.

TNT, RDX, and PETN powders were obtained from a Korean company, PNL global. Since both RDX and PETN cannot be dissolved in water, acetone (Sigma Aldrich) was used as solvent. TNT was dissolved in water as well as in hexane. To obtain 1 mM of TNT, 9.08 mg was dissolved in 40 mL of deionized (DI) water. We also dissolved 9.08 mg of TNT in 40 mL of hexane to obtain a 1 mM solution. Then, 1 mM solutions of RDX and PETN were obtained by dissolving 8.88 mg and 12.64 mg in 40 mL acetone, respectively. Then, the solutions were diluted to picomolar (pM) concentrations. Gold nanogap substrates were plasma treated using inductively coupled plasma (ICP) (Atmospheric Process Plasma, APP Co., Ltd. Seoul, South Korea) with 50 W RF power and O_2_ flow of 20 sccm for 2 min. The condition was chosen not to damage nanogap substrates by the increased RF power and plasma treating time. With the oxygen plasma treatment on the substrate, we observed the change of the surface state from hydrophobic to hydrophilic. This is consistent with the previous result reported in [38], where the contact angle of water was reduced to less than 5°.

Raman scattering measurements were carried out using a LabRAM HR-800 UV-Visible-NIR (Horiba Jobin Yvon) equipped with a multichannel air-cooled CCD detector. The excitation source was a 785 nm laser with an incident power of ≈0.1 mW. An objective lens of 50× (N.A.: 0.75) with a laser spot diameter of 1.28 µm was employed for the SERS measurement with an integration time of 10 s. Then, 2 µL solution was dropped on the nanogap sample of 0.5 cm × 0.5 cm size and left to dry under the normal atmospheric condition. The SERS spectra were measured at random locations on the sample under the assumption that explosive molecules are evenly distributed [39].

## 3. Results and Discussion

Figure 2b shows an SEM image of an Au nanogap substrate. The nanosphere diameter was estimated to be ≈85 ± 20 nm, while the gap width is ≈10 nm. The electric field around the nanogap was calculated utilizing a finite-difference time-domain (FDTD) software, FDTD Solutions (Lumerical Inc., Vancouver, Canada,). Physical dimensions used for the simulation were 4 µm, 4 µm, and 1.5 µm in the x, y, and z directions, respectively. The mesh size around the nanogap structure was set to 1 nm in all directions. Boundary conditions were set to perfectly matched layers (PMLs), and the light source used for the simulation was total field/scattered field (TFSF). As seen in Figure 2c, the strong enhancement of electric field was observed in the gap (corresponding to the hot zone) between the nanosphere and metal plane. The center wavelength of the nanogap resonance was estimated to be ≈780 nm for the employed nanogap substrates.

**Figure 3:**
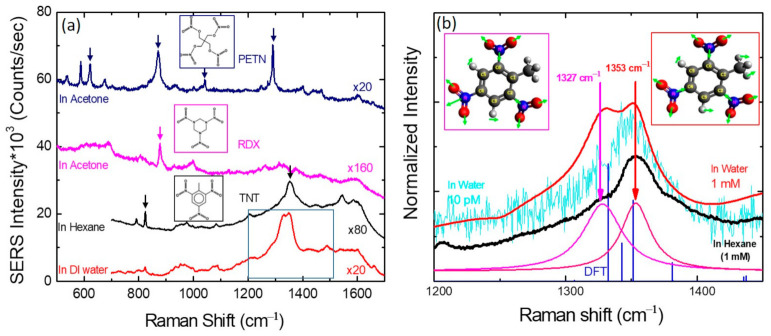
*(***a**) SERS spectra from PETN, RDX, TNT/water, and TNT/hexane. The concentrations of all four samples are the same, 1 mM. The geometry of each molecule is given in the inset. (**b**) Symmetric NO_2_ modes of TNT molecules in the SERS spectra compared with the DFT simulation results (blue). The vibrational directions (green arrows) of the atoms are shown in the inset.

Figure 3a shows the SERS spectra of PETN, RDX dissolved in acetone, and of TNT in hexane as well as in DI water. The measured SERS peaks match well with those reported in the literature [40,41,42,43,44]. Vibrational modes in PETN include 620 cm^−1^ (ONO_2_ rocking), 870 cm^−1^ (O-N stretching), 1042 cm^−1^ (CH_2_ torsion and C-C bending), and 1290 cm^−1^ (NO_2_ symmetric stretching). A characteristic RDX peak was found at 877 cm^−1^ (C-N-C stretching). The molecular structures of the explosives are included in the figure. As seen in Figure 3a, symmetric NO_2_ stretching vibration at 1353 cm^−1^ is the dominant feature in the SERS spectra of TNT molecules irrespective of the solvent used. In order to examine the SERS peak at around 1350 cm^−1^, in Figure 3b, we show the enlarged SERS spectra for TNT/DI water with two TNT concentrations of 1 mM and 10 pM as well as 1 mM TNT/hexane. When we resolved the SERS peak for 1 mM at around 1350 cm^−1^, two Gaussian peaks were located at 1327 cm^−1^ and 1353 cm^−1^, which are very close to the theoretically expected SERS peaks obtained from Density Functional Theory (DFT) simulations (Orca 4.2.0) using the B3LYP method with 6-311++G (d,p) basis function (blue lines). The difference between the two NO_2_ symmetric stretching modes is the relative vibrational phase of the three nitrogen atoms in the TNT molecules. The three nitrogen atoms attached to carbon atoms C2, C4, and C6 vibrate inward and outward “in phase” corresponding to the 1353 cm^−1^ peak, whereas the nitrogen atom attached to C6 vibrates “out of phase” with respect to the nitrogen atoms attached to C2 and C4, giving rise to the peak at 1327 cm^−1^. The molecular structure of TNT is shown in the inset where the arrows represent the vibrational directions of the atoms. In the Supplementary Materials Videos S1 and S2, we show animations for the two vibrations. We also show in Supplementary Video S3 an animation of the C-N-C stretching mode of RDX obtained from DFT simulations.

**Figure 4:**
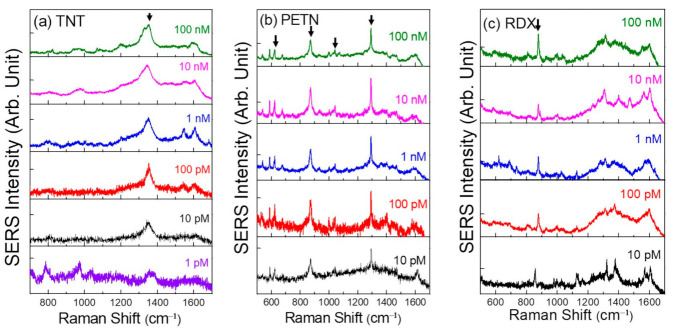
The SERS spectra of explosive materials at various mole concentrations. (**a**) SERS spectra of TNT with different concentrations. SERS spectra of (**b**) PETN and (**c**) RDX.

Figure 4a shows the SERS spectra of TNT/DI water concentrations down to 1 pM. The main characteristics peaks of PETN are located at 870 cm^−1^ (O-N stretching mode) and 1290 cm^−1^ (NO_2_ symmetric stretching), whereas for RDX, the dominant peak is at 877 cm^−1^ (C-N-C stretching mode). As seen in Figure 4b,c, all the dominant SERS peaks are clearly visible even for the low concentration of 10 pM.

We note here that the mass of TNT in 2 µL solution for 10 pM is only ≈5 fg, and the number of molecules corresponds to only ≈1 × 10^7^ on a sample piece with 5 mm × 5 mm size. Considering that the spot size of a laser beam is 1.28 µm (objective 50×) at 785 nm, the number of TNT explosive molecules for 10 pM corresponds to ≈1 molecule within the detection area. Thus, the sensitivity is significantly improved as compared to previous reports [11,20].

**Figure 5:**
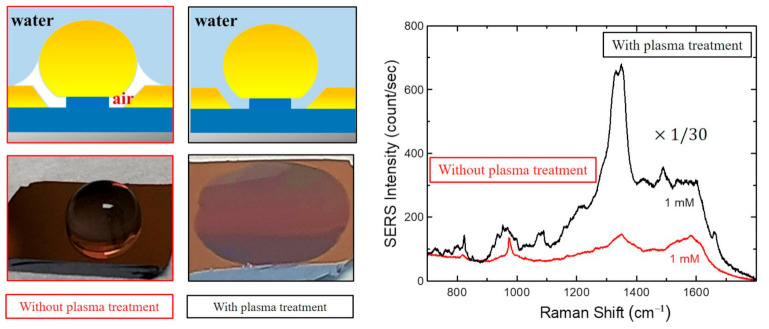
SERS spectra measured from Au nanogap samples with the plasma treatment (black), prior to TNT coating, and without plasma treatment (red) for TNT/water. The concentration is 1 mM for both samples, and the measurement conditions are the same as described in the Method section. Images at the left show the hydrophobic and hydrophilic surface states before and after the plasma treatment, respectively.

Since Ag nanogap substrates deteriorate quickly in humid environments, Au nanogap substrates were chosen in this work. In Figure 5, we compare the SERS spectra of TNT/water solution on Au nanogap substrates with and without plasma treatment. As seen, the SERS intensity was significantly enhanced by the plasma treatment. The enhancement was attributed to the surface change from hydrophobic to hydrophilic. Such surface change by plasma treatment is consistent with the previous report [45]. With the hydrophilic surface, TNT aqueous solution can penetrate into the gap and TNT molecules can be adsorbed in the gap area (hot spot), resulting in high sensitivity.

## 4. Conclusions

We performed SERS measurements using Au nanogap substrates and achieved outstanding detection limit of only a few molecules for each explosive. The detection of explosive compounds was possible down to as low as a picomolar range of concentration. The enhancement of SERS intensity for TNT in DI water with the plasma treatment on the Au nanogap substrate is attributed to the surface change from hydrophobic to hydrophilic. Variations in SERS spectra observed for different explosives play a crucial role in distinguishing and identifying different nitro-based compounds. This is an important advantage over the fluorescence quenching methods using various polymers and quantum dots. Our results attest that the present cost-effective SERS substrate is very sensitive for nitro compounds and useful for practical applications.

## Data Availability

Data generated for this study can be found here: https://drive.google.com/drive/folders/1WK5Ko5Zjj6Jj-OUiV6l95nokKm_5JYii?usp=sharing (accessed on 15 July 2021).

## Supplementary Materials

The following are available online at https://www.mdpi.com/article/10.3390/s21165567/s1. Animations of the NO_2_ symmetric vibrations of a TNT molecule corresponding to the peaks at 1327 cm^−1^ (Video SI) and 1353 cm^−1^ (Video S2). Video S3: Animation of C-N-C stretching mode of α-RDX corresponding to the peak at 877 cm^−1^. Animations can be found at: https://drive.google.com/drive/folders/1WK5Ko5Zjj6Jj-OUiV6l95nokKm_5JYii?usp=sharing (accessed on 15 July 2021)

## References

[1] Dasary S.S.R., Singh A.K., Senapati D., Yu H., Ray P.C. (2009). Gold Nanoparticle Based Label-Free SERS Probe for Ultrasensitive and Selective Detection of Trinitrotoluene. J. Am. Chem. Soc..

[2] van Dillewijn P., Couselo J.L., Corredoira E., Delgado A., Wittich R.-M., Ballester A., Ramos J.L. (2008). Bioremediation of 2,4,6-Trinitrotoluene by Bacterial Nitroreductase Expressing Transgenic Aspen. Environ. Sci. Technol..

[3] Ewing R.G., Atkinson D.A., Eiceman G.A., Ewing G.J. (2001). A Critical Review of Ion Mobility Spectrometry for the Detection of Explosives and Explosive Related Compounds. Talanta.

[4] Yang J.S., Swager T.M. (1998). Porous Shape Persistent Fluorescent Polymer Films: An Approach to TNT Sensory Materials. J. Am. Chem. Soc..

[5] Sylvia J.M., Janni J.A., Klein J.D., Spencer K.M. (2000). Surface-Enhanced Raman Detection of 2,4-Dinitrotoluene Impurity Vapor as a Marker To Locate Landmines. Anal. Chem..

[6] Laurence T.A., Braun G., Talley C., Schwartzberg A., Moskovits M., Reich N., Huser T. (2009). Rapid, Solution-Based Characterization of Optimized SERS Nanoparticle Substrates. J. Am. Chem. Soc..

[7] Camden J., Dieringer J.A., Zhao J., Van Duyne R.P. (2008). Controlled Plasmonic Nanostructures for Surface-Enhanced Spectroscopy and Sensing. Acc. Chem. Res..

[8] He X., Wang H., Li Z., Chen N., Liu J., Zhang Q. (2015). Ultrasensitive SERS detection of trinitrotoluene through capillarity-constructed reversible hot spots based on ZnO–Ag nanorod hybrids. Nanoscale.

[9] Liyanage T.U.L.H., Rael A., Shaffer S., Zaidi S., Goodpaster J.V., Sardar R. (2018). Fabrication of a self-assembled and flexible SERS nanosensor for explosive detection at parts-per-quadrillion levels from fingerprints. Analyst.

[10] Huang Y., Liu W., Gong Z., Wu W., Fan M., Wang D., Brolo A.G. (2020). Detection of Buried Explosives Using a Surface-Enhanced Raman Scattering (SERS) Substrate Tailored for Miniaturized Spectrometers. ACS Sens..

[11] Ko H., Chang S., Tsukruk V. (2009). Porous Substrates for Label-Free Molecular Level Detection of Nanoresonant Organic Molecules. ACS Nano.

[12] Shen Z., Su L., Shen Y.-C. (2016). Vertically-oriented nanoparticle dimer based on focused plasmonic trapping. Opt. Express.

[13] Kneipp K., Kneipp H., Itzkan I., Dasari R.R., Feld M.S. (1999). Ultrasensitive Chemical Analysis by Raman Spectroscopy. Chem. Rev..

[14] Kneipp K., Wang Y., Kneipp H., Perelman L.T., Itzkan I., Dasari R.R., Feld M.S. (1997). Single Molecule Detection Using Surface-Enhanced Raman Scattering (SERS). Phys. Rev. Lett..

[15] Nie S., Emory S. (1997). Probing Single Molecules and Single Nanoparticles by Surface-Enhanced Raman Scattering. Science.

[16] Hankus M.E., Stratis-Cullum D., Pellegrino P.M. Surface enhanced Raman scattering (SERS)-based next generation commercially available substrate: Physical characterization and biological application. Proceedings of the Biosensing and Nanomedicine IV.

[17] McNay G., Eustace D., Smith W.E., Faulds K., Graham D. (2011). Surface-Enhanced Raman Scattering (SERS) and Surface-Enhanced Resonance Raman Scattering (SERRS): A Review of Applications. Appl. Spectrosc..

[18] Mosier-Boss P.A. (2017). Review of SERS Substrates for Chemical Sensing. Nanomaterials.

[19] Kneipp K., Wang Y., Dasari R.R., Feld M.S., Gilbert B.D., Janni J., Steinfeld J.I. (1995). Near-infrared surface-enhanced Raman scattering of trinitrotoluene on colloidal gold and silver. Spectrochim. Acta Part A Mol. Biomol. Spectrosc..

[20] Botti S., Almaviva S., Cantarini L., Palucci A., Puiu A., Rufoloni A. (2013). Trace level detection and identification of nitro-based explosives by surface-enhanced Raman spectroscopy. J. Raman Spectrosc..

[21] Demeritte T., Kanchanapally R., Fan Z., Singh A.K., Senapati D., Dubey M., Zakar E., Ray P.C. (2012). Highly efficient SERS substrate for direct detection of explosive TNT using popcorn-shaped gold nanoparticle-functionalized SWCNT hybrid. Analyst.

[22] Chen N., Ding P., Shi Y., Jin T., Su Y., Wang H., He Y. (2017). Portable and Reliable Surface-Enhanced Raman Scattering Silicon Chip for Signal-On Detection of Trace Trinitrotoluene Explosive in Real Systems. Anal. Chem..

[23] Zhou H., Zhang Z., Jiang C., Guan G., Zhang K., Mei Q., Liu R., Wang S. (2011). Trinitrotoluene Explosive Lights up Ultrahigh Raman Scattering of Nonresonant Molecule on a Top-Closed Silver Nanotube Array. Anal. Chem..

[24] To K.C., Ben-Jaber S., Parkin I.P. (2020). Recent Developments in the Field of Explosive Trace Detection. ACS Nano.

[25] Alexander T.A., Le D.M. (2007). Characterization of a commercialized SERS-active substrate and its application to the identification of intact Bacillus endospores. Appl. Opt..

[26] Hadjiivanov K.I., Panayotov D.A., Mihaylov M.Y., Ivanova E.Z., Chakarova K.K., Andonova S.M., Drenchev N.L. (2021). Power of Infrared and Raman Spectroscopies to Characterize Metal-Organic Frameworks and Investigate Their Interaction with Guest Molecules. Chem. Rev..

[27] Clarkson J., Smith W., Batchelder D.N., Smith D., Coats A.M. (2003). A theoretical study of the structure and vibrations of 2,4,6-trinitrotolune. J. Mol. Struct..

[28] Zapata F., López-López M., García-Ruiz C. (2015). Detection and identification of explosives by surface enhanced Raman scattering. Appl. Spectrosc. Rev..

[29] Primera-Pedrozo O.M., Jerez-Rozo J.I., De La Cruz-Montoya E., Luna-Pineda T., Pacheco-Londono L.C., Hernandez-Rivera S.P. (2008). Nanotechnology-Based Detection of Explosives and Biological Agents Simulants. IEEE Sensors J..

[30] Hernández-Rivera S.P., Briano J.G., De La Cruz-Montoya E., Pérez-Acosta G.A., Jeréz-Rozo J.I. (2009). Enhanced Raman Scattering of Nitroexplosives on Metal Oxides and Ag/TiO2 Nanoparticles. ACS Symp. Ser..

[31] Rozo J.I.J., Chamoun A.M., Peña S.L., Hernández-Rivera S.P. Enhanced Raman scattering of TNT on nanoparticle substrates: Ag colloids prepared by reduction with hydroxylamine hydrochloride and sodium citrate. Proceedings of the Defense and Security Symposium.

[32] Farrell M.E., Holthoff E.L., Pellegrino P.M. Next-generation surface-enhanced Raman scattering (SERS) substrates for hazard detection. Proceedings of the SPIE Defense.

[33] Echols R.T., Christensen M.M., Krisko R.M., Aldstadt J.H. (1999). Selective Determination of TNT in Soil Extracts by Sequential Injection Spectrophotometry. Anal. Chem..

[34] Dick L.A., McFarland A.D., Haynes C., Van Duyne R.P. (2002). Metal Film over Nanosphere (MFON) Electrodes for Surface-Enhanced Raman Spectroscopy (SERS): Improvements in Surface Nanostructure Stability and Suppression of Irreversible Loss. J. Phys. Chem. B.

[35] Adhikari S., Kim M., Lee J., Jang Y., Hong C., Jeong Y., Baek J., Lee J., Lee S., Kim J. (2021). Six Inch Uniform and High Enhancement SERS Substrate with Hole-sphere Gold Nanogaps for Quantitative Measurements.

[36] Kim J., Lee C., Lee Y., Lee J., Park S., Park S., Nam J. (2021). Synthesis, Assembly, Optical Properties, and Sensing Applications of Plasmonic Gap Nanostructures. Adv. Mater..

[37] Wang L., Kafshgari M.H., Meunier M. (2020). Optical Properties and Applications of Plasmonic-Metal Nanoparticles. Adv. Funct. Mater..

[38] Yamamoto M., Matsumae T., Kurashima Y., Takagi H., Suga T., Itoh T., Higurashi E. (2019). Comparison of Argon and Oxygen Plasma Treatments for Ambient Room-Temperature Wafer-Scale Au–Au Bonding Using Ultrathin Au Films. Micromachines.

[39] Almaviva S., Botti S., Cantarini L., Fantoni R., Lecci S., Palucci A., Puiu A., Rufoloni A. (2013). Ultrasensitive RDX detection with commercial SERS substrates. J. Raman Spectrosc..

[40] Tuschel D.D., Mikhonin A.V., Lemoff B.E., Asher S.A. (2010). Deep Ultraviolet Resonance Raman Excitation Enables Explosives Detection. Appl. Spectrosc..

[41] Gruzdkov Y.A., Gupta Y.M. (2001). Vibrational Properties and Structure of Pentaerythritol Tetranitrate. J. Phys. Chem. A.

[42] Infante-Castillo R., Pacheco L., Hernández-Rivera S.P. (2010). Vibrational spectra and structure of RDX and its 13C- and 15N-labeled derivatives: A theoretical and experimental study. Spectrochim. Acta Part. A Mol. Biomol. Spectrosc..

[43] Miao M.S., Dreger Z.A., Winey J.M., Gupta Y.M. (2008). Density Functional Theory Calculations of Pressure Effects on the Vibrational Structure of α-RDX. J. Phys. Chem. A.

[44] Liu Y., Perkins R., Liu Y., Tzeng N. (2017). Normal mode and experimental analysis of TNT Raman spectrum. J. Mol. Struct..

[45] Zhu J., Zangari G., Reed M.L. (2011). Tailoring the Wetting Properties of Surface-Modified Nanostructured Gold Films. J. Phys. Chem. C.

